# Hardware Trojan Mitigation Technique in Network-on-Chip (NoC)

**DOI:** 10.3390/mi14040828

**Published:** 2023-04-08

**Authors:** Musharraf Hussain, Naveed Khan Baloach, Gauhar Ali, Mohammed ElAffendi, Imed Ben Dhaou, Syed Sajid Ullah, Mueen Uddin

**Affiliations:** 1 Faculty of Computer Science, The University of Lahore, Islamabad Campus, Islamabad 44000, Pakistan; 2 Faculty of Computer Engineering, University of Engineering and Technology, Taxila 47050, Pakistan; 3 EIAS Data Science and Blockchain Lab, College of Computer and Information Sciences, Prince Sultan University, Riyadh 11586, Saudi Arabia; 4 Department of Computer Science, Hekma School of Engineering, Computing, and Informatics, Dar Al-Hekma University, Jeddah 34801, Saudi Arabia; 5 Department of Computing, University of Turku, 20500 Turku, Finland; 6 Higher Institute of Computer Sciences and Mathematics, Department of Technology, University of Monastir, Monastir 5000, Tunisia; 7 Department of Information and Communication Technology, University of Agder (UiA), N-4898 Grimstad, Norway; 8 College of Computing and IT, University of Doha for Science and Technology, Doha 24449, Qatar

**Keywords:** Trojan mitigation, hardware Trojan mitigation, network-on-chip

## Abstract

Due to globalization in the semiconductor industry, malevolent modifications made in the hardware circuitry, known as hardware Trojans (HTs), have rendered the security of the chip very critical. Over the years, many methods have been proposed to detect and mitigate these HTs in general integrated circuits. However, insufficient effort has been made for hardware Trojans (HTs) in the network-on-chip. In this study, we implement a countermeasure to congeal the network-on-chip hardware design in order to prevent changes from being made to the network-on-chip design. We propose a collaborative method which uses flit integrity and dynamic flit permutation to eliminate the hardware Trojan inserted into the router of the NoC by a disloyal employee or a third-party vendor corporation. The proposed method increases the number of received packets by up to 10% more compared to existing techniques, which contain HTs in the destination address of the flit. Compared to the runtime HT mitigation method, the proposed scheme also decreases the average latency for the hardware Trojan inserted in the flit’s header, tail, and destination field up to 14.7%, 8%, and 3%, respectively.

## 1. Introduction

The complexity of the chip design has drastically increased as a result of the decrease in transistor feature sizes and the replacement of traditional planar CMOS technology with the multigate transistor, commonly known as FinFet technology. The most recent Intel processor, released in the fourth quarter of 2022, is built using a 10 nm FinFET transistor and has 24 cores [1]. The transistor count for contemporary processors exceeds 6 billion.

**Figure 1:**
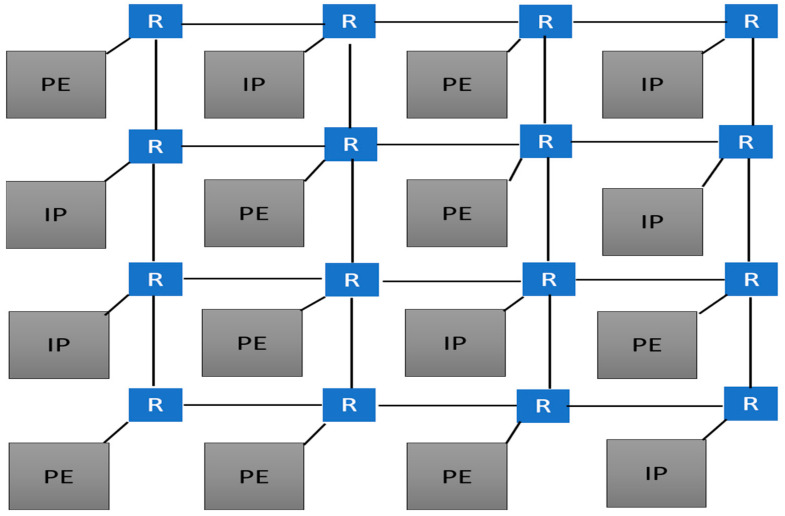
Mesh NoC architecture.

IP-based design is an invertible methodology to deal with the high integration levels and decreasing time-to-market. Additionally, in the SoC paradigm, in the nanometer design, the effects of interconnects dominate the design’s performance [2]. The networks on chip (NoC) has been proposed as an alternative way to solve the communication bottlenecks in the multi-core and multi-processor design [3]. There are two popular architectures for the NoC: Torus and mesh [4]. The mesh architecture consumes less power than the Torus NoC. Figure 1 depicts a 4 × 4 NoC architecture using mesh topology. The architecture is composed of both processing elements and IPs.

There are numerous hardware threats available, and hardware Trojan is one the most visible threats, since the majority of companies outsource the fabrication, testing, and assembly of the chip. As a result, outsourcing different chip component processes and hardware security has become very challenging [5]. Hardware Trojan is the modification in the circuitry of the chip. At the time of fabrication, the ion beams can be used to insert the dormant Trojan, which may disturb the functional behavior of the chip [6]. These kinds of attacks are called multilevel attacks, which may degrade the overall functionality of the chip. Taking the crypto module as an example, it is clear that the attacks posed on the crypto module are more dangerous than a single adverse attack [7]. As an example, suppose an adversary attacks the AES module by inserting faults in it to obtain the cyber key [8]. This can be only maliciously modified by an adversary who is a part of the conspiracy. Trojans can be inserted locally or distributed throughout the network. The payload and the trigger part of the Trojan can be digital or analog, as malicious modification can occur when the temperature of the chip changes [9].

The payload circuit defines the threat of the Trojan hardware, which may be a denial of service (DoS), alter the chip’s normal operation, or grant privileges to the attacker access to the classified memory gap [10]. The Semiconductor Research Corporation (SRC), a research community for hardware, has reviewed that fewer detection methods of the hardware Trojans are present, which is creating a challenging environment for hardware security [11]. Existing methods for detecting hardware Trojans can be divided into two main categories: destructive and non-destructive methods. The malicious circuits in the chips containing the hardware Trojan will harm to the overall system [12]. Network-on-chip is a concept in which reliable and high-performance routers for multiple IP cores are equipped with single silicon chips [13]. Despite the severity of this threat, current detection methods for hardware Trojans are limited and can be classified into two categories: invasive methods and non-invasive methods. This gap in existing detection methods highlights the need for a more effective solution. Our proposed solution aims to fill this gap by utilizing non-invasive algorithms to detect hardware Trojans at an early stage, before it can cause significant damage to the system. Our approach is non-invasive and can be applied to various types of hardware Trojans. Furthermore, our solution can also detect distributed Trojans, which are often more difficult to detect than local Trojans. Our proposed approach aims to enhance hardware system security and protect against the ever-increasing threat of hardware Trojan attacks.

The following sections of the paper are organized as follows. In Section 2, we discuss how Trojan hardware could be inserted into the NoC. In Section 3, we summarize related hardware Trojan detection and mitigation methods for the network-on-chip and our contribution to them. The proposed technique is discussed in Section 4, which encompasses the hardware Trojan detection and network-on-chip integrity check, and the unique and unpredictable permutation selector signal. Section 6 discussed the experimental results. Finally, Section 6 conclude our research with future work.

## 2. Hardware Trojan Insertion Method in NoCs

### 2.1. Attacking Scenario

**Figure 2:**
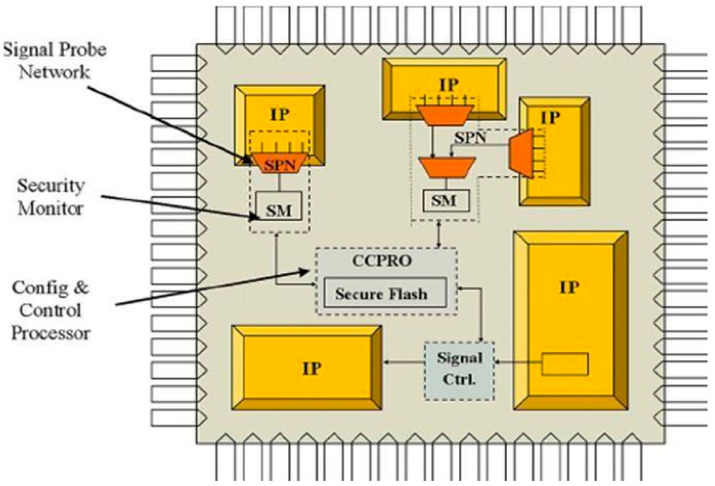
Insertion of a hardware Trojan.

HTs are specifically created to deliver numerous attacks, including DoS attacks, attacks aimed at leaking information, attacks that maliciously manipulate data, or attacks that degrade system performance. Through manipulations in the design process, HTs can be placed next to the RTL description [14], directly into the gate-level netlist, which can ultimately lead to logical assaults taking place in the system. Recent work has focused on detecting hardware Trojans using various techniques, including power analysis, functional testing, and side-channel analysis. However, the proposed approach using electro-optical frequency mapping images for detecting hardware Trojans represents a novel and promising research direction in the future [15]. While the designs and arrangements can be altered and customized to include an HT at the prototyping/fabrication stage by modifying the characteristics of the contained circuitry, one of those characteristics could be rescheduling [16]. As shown in Figure 2, the objective of the countermeasure that is being considered for this attempt is to eliminate the HTs that were introduced into the NoC by dishonest and fraudulent team members who were employed by the NoC design house or by a third-party company that was responsible for the integration of multiple processors (MPSoC). Security monitors (SMs) are used to test the functionalities of the system on the chip to enable the real-time functionality monitoring system [17]. SM-based monitoring systems are used to develop finite state machines (FSMs), whose function is to check the behavior of the signal of interest. These signals feed the signal probe network (SPN) [18]. Security monitors are configured in such a way that they cannot interfere with the functionality of the other security monitors. When any deviation from the normal operation is detected, the normal operation and trigger circuitry operation are both performed simultaneously to examine these Trojan behaviors [3].

### 2.2. NoC Baseline Architecture

**Figure 3:**
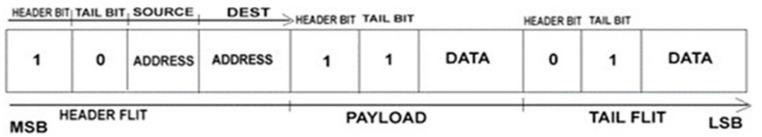
Packet format that is utilized for the proposed work.

Flits are the fundamental building blocks that control the flow transfer in an NoC and include the header flit, the tail flit, and the many payload flits. The initial bit indicates the header flit, and the high logic of this bit indicates the presence of header flits. The next bit indicates the tail flit, and the high logic of this bit indicates the tail flit’s entrance. The remaining flits contain information such as routing protocols or data bits, source identifiers, or destination identifiers [19]. The source address flit, destination address flit, and information flit type control how the NoC router sends flits across numerous hop routing paths. The packet layout is shown in Figure 3. For this study, we will use a simplified version of the packet format that is used by Intel’s NoC [20]. Malicious amendments to the header flit will possibly result in damage to the integrity of the data, and may also cause the misrouting of the packets, or the flit may be lost altogether. The misrouted packets may violate the deadlock-free routing rules and cause a deadlock in the network. Deadlock, live-lock, and flit letdown result in bandwidth depletion [21].

### 2.3. Trojan Attacks in NoCs

When HTs are inserted in NoCs based on MPSoCs, they may steal confidential information and hijack more easily in the processing element compared to NoC routers [22]. The primary goal of this work is to strengthen the network-on-chip design, which is why we are considering the types of HT attacks that could result in denial-of-service attacks in the NoC routers. Due to DoS attacks which may cause bandwidth depletion, live-lock, and misroute in the network-on-chip as well as the deadlock in NoC. The HTs in the NoC may also alter the destination/source address of the packets and as well as the type of flit. If the destination HT is inserted, it may change the flit’s destination and lead it to another destination. When the header or the tail HTs are inserted into the NoC, the flit may retain control of the router until some of the acts retune. Details of the HT are discussed in [23].

## 3. Related Work

The security of the NoC has received ample attention in recent decades. A recent survey summarizes countermeasure techniques to address five classes of attacks: eavesdropping, spoofing and data integrity, denial of service, buffer-overflow and memory extraction, and side channel.

A comprehensive survey has been reported on the use of a physically unclonable function (PUF) for authenticating and generating cryptographic keys [24]. Compromised NoCs detection methods at the firmware level are discussed in [25]. To aggravate the attacks, the network-on-chip employs the encryption authentication framework method [26]. The security of the NoC is compromised by HTs’ network interfaces or modules that are directly connected to the IP cores of the chip [27]. Testing of the memory address method to prevent the unsecured zone from communicating with the secure zone is presented in [28]. The method used for memory protection, which is based on the lookup table, may provide authentication against any malicious packets in the NoC [29]. To prevent the packets from being transferred from the secure zone to the secure zone, communication barriers are used [30]. Keykeepers and security wrappers are used to encrypt public and private keys at the core level to prevent cryptography attacks at the core level. The existing method for HTs considers security at the network interfaces (NIs) which may be able to prevent illegal memory addresses, as well as packet communication at NIs, but there is no method to mitigate the depletion of the bandwidth in the NoC [31]. Certain methods of the hardware Trojans are built into the NoC microarchitecture. To control denial-of-service attacks when an HT has been inserted in the NoC, Wange et al. [32] proposed a method sets the upper throughput limit at the switch allocators of the NoC. In order to prevent low-security flits from exceeding the static limits of the security channels, which have low limits, some control is given to the output and input arbiters [33]. A user-defined function named “counter” is used to calculate the throughput of the flits, and if the counter is modified maliciously, then the defense of the security method will fail.

Baron et al. [22] used two levels of security wrappers to address the camouflaged and denial-of-service attacks in the NoC system. One wrapper was used to compare the destination address of the ongoing packets from the router to check the legality of the destination address of the packets. Another wrapper was used for counting the number of cycles when the packet is inserted into the NoC so that it can postpone the other packet’s injection if a HT is present in the network. Thus, by studying this approach, a packet may be blocked from being transferred from the non-secure zone to the secure zone; however, a person with access to the security wrapper can add a malicious packet to the network. This method also has the drawback of latency and area overhead. Because of the malicious modification made in the NoC, the hardware Trojan that was implanted into the network-on-chip will be able to obtain information leakage, unauthorized memory access, and denial-of-service attack due to incorrect path routing, live-lock, or deadlock. Additionally, the hardware Trojan will be able to obtain denial-of-service attack. The existing methods used for detecting hardware Trojans are designed for general ICs [34]. However, there are few active methods that may strengthen the network-on-chip design. In this paper, we propose a method for protecting the network-on-chip against hardware Trojan tampering. In contrast to the existing approaches, the proposed method includes the full features of the network-on-chip: the parallel transmission channels, high scalability, and high modularity when we strengthen the NoC against hardware Trojans attacks.

Kim et al. came up with a method in which the restricting address register would be applied to the address decoder. This would prevent bus masters and slaves that had been maliciously modified from accessing the restricted address range [35]. The drawback of this method is that the restricted address is not fully able to be seen by the master bus and can be changed from the external port, thus it may readily undergo malicious modification by attackers. Kim et al. [28] presented a secret code method in which the authorized personnel may also easily find the packets which have been modified maliciously; however, they do not obtain the risk and cost of the authenticated code. For the bus arbiter, [36] uses a time-based counter and statistical analyses to find the uncertain bus mastership; however, the methods based on the time required to detect the HTs is considered wasted when the Trojan is triggered. This is because it will deplete the packet when a maliciously modified packet travels through the network-on-chip.

Previous work presented in [37] on the assessment of the range to access the memory was regarded the key method that could be considered secure. However, these methods have the drawback of checking the range of the memory by itself, which requires protection, and when the packets pass to the memory range, the packets at the routers and NI are not protected. Furthermore, there is a chance that the router and the NI can be negotiated during the route computation stage. Because the memory address is stored in the output buffers, a person can quickly change it. There are some methods for detecting HTs that are present in the NIs but not present in the routers of the network-on-chip communication [38]. As we know, every router has five connection nodes with its neighboring router nodes, but only the network interface has a connection with the IP core and a link to the router. As a result, we can conclude that the router is more prone to denial-of-service attacks compared to NIs.

Artificial Intelligence (AI) is another field in which researchers attempt to discover new ways to gain insight and detect and eliminate anomalies in networks. Shah, Fadia et al. presented a method using AI as a service (AIaaS). The immoral content is detected and eliminated in this method, but the same concept can be used to detect and eliminate the NoC’s attack [39]. Qureshi, Muhammad Bilal et al. discussed the existing encryption techniques for smart system data security. These techniques can be used to secure the data stored on NoCs [40].

The authors of [39] developed a machine learning-based approach for locating IPs responsible for DOS attacks. The technique uses a router with feature collection capabilities and a perceptron unit.

The shortcomings of previous methods were that they assumed the routers could be trusted, and the flows of flits that occurred between the routers did not receive complete coverage. As a result, we relied on MAC to cover the flows of flits that occurred within the routers themselves. The newly proposed method gets around the drawbacks of the older methods and offers advantages in terms of flit integrity, flit permutation, and MAC.

## 4. Method Proposed for HT Mitigation in NoC Routers

### 4.1. Message Authentication Code (MAC)

MAC is a message authentication procedure that is based on well-established cryptographic primitives, i.e., symmetric key cryptography. To undertake MAC processes, the sender and receiver share a symmetric key K. MAC mostly refers to an encrypted checksum generated by the underlying message and sent along with it to ensure that the message transfer is authentic. At the sender side, the MAC algorithm uses the message to be sent and a secret key K and produces a MAC value which is sent along with the message. MAC, like HASH, compresses a large input to an arbitrarily fixed-length output. However, a significant difference between MAC and HASH is that the MAC algorithm uses a secret key during the compression, which occurs after the sender sends the message along with that MAC value. At this stage, we consider the message to be clear and that there are no errors; however, if we require a confidential MAC, then we must encrypt the message. The message and secret key are used to regenerate the MAC value on the receiver side. Then, the MAC received at the receiver side and regenerated MAC at the receiver are compared; if they both are equal, the receiver will accept the message and be ensured that the message was sent from an authentic sender. If the received MAC and the regenerated MAC calculated at the receiver side do not match, the receiver will not accept the message and consider it faulty.

### 4.2. HMAC

A hash function is a type of function that maps an arbitrary size of data to a fixed size of data. The value which is returned by a hash function is known as a hash value, a hash code, or, simply, hash. One use of the hash function is the hash table, which is widely used in different computer software for searching data rapidly. The primary function of the hash function is that it speeds up the database or table search to detect duplicated data in a large file. Finding similar stretches in DNA sequences is an example of a hash function. A hash function is also useful in cryptography. The cryptographic hash function is responsible for ensuring that the input data will match an already stored hash value. If the input data is unknown, then it is challenging to construct a hash value for it because it is more difficult to reconstruct the hash value for unknown input, as this is used for assuring the integrity of the transmitted data, and this is the building blocks of HMACs because HMAC provides message authentication.

HMAC (keyed-hash-based message authentication code) is a type of message authentication code (MAC) containing a cryptographic hash function and a secret key for cryptography. HMAC is mainly used for both data integrity and message authentication. The strength of the HMAC depends on the length of the underlying hash function, the size of the hash output, and the size and quality of the key used.

To generate HMAC, it must first pass two hash computations. The secret key generates both the inner and outer secret keys. The first pass of this algorithm uses the secret key and message to generate an internal hash value; the second pass uses the inner hash value and the outer key to generate the final HMAC. This algorithm is resistant to length, extension, and data integrity attacks.

### 4.3. Implementation of HMAC

**Figure 4:**
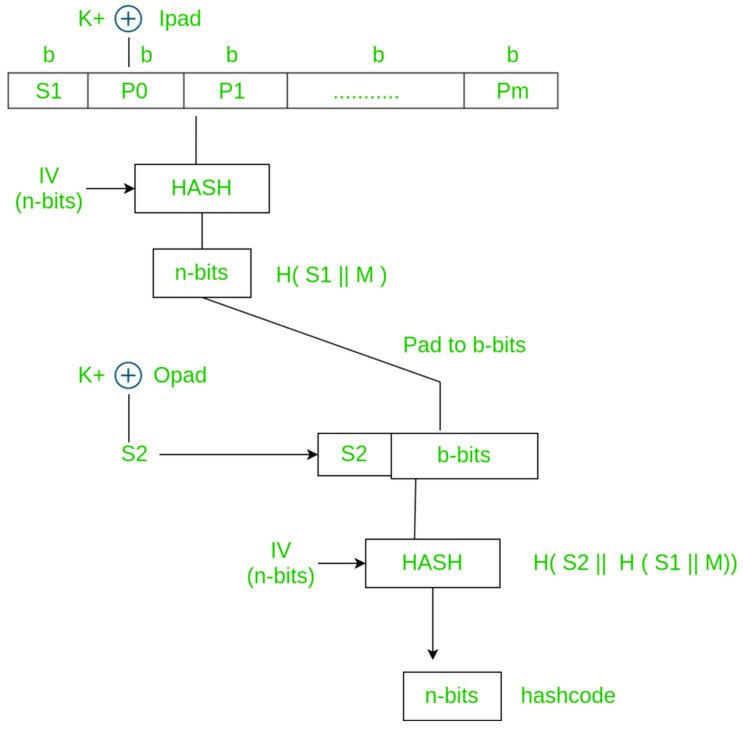
Representation of the HMAC algorithm.

Figure 4 explains how the HMAC algorithm is implemented.

In Figure 4 above clearly shows that this algorithm first checks the length of the key; if the length of the key is greater than the block size, then we shrink the size of the key by applying the hash function. If the key size is less than that of the block size, we enlarge the key size by adding 0’s to the key. Following that, we calculate the outer key (S2) and the inner key (S1) used in the HMAC algorithm. Furthermore, we return H (S2 ∥ H (S1 ∥ M)), which means that we return a value in which the inner key (S1) and message are concatenated, and then we apply the hash function to it, and then this result is concatenated with the outer key (S2). Finally, we again apply the hash function to the overall result and return that value from the HMAC algorithm. Hence, the integrity of the message can be preserved by applying these types of restrictions.

## 5. Overview of the Proposed Scheme

**Figure 5:**
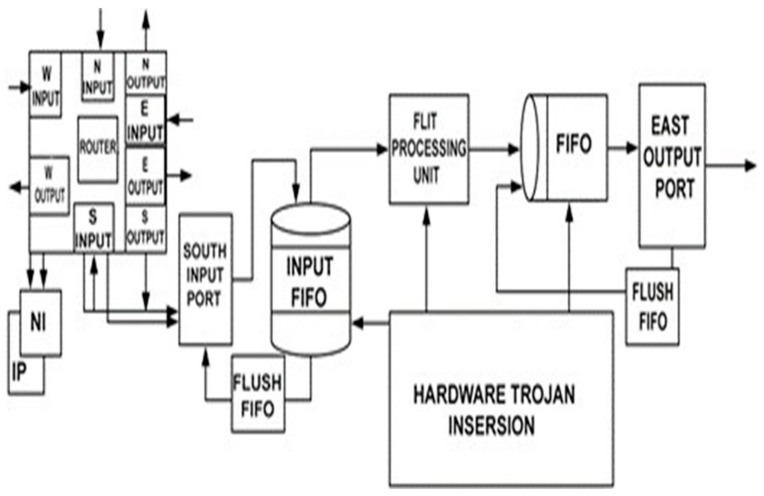
High-level view of the proposed technique.

The proposed method aims to detect and mitigate HTs that are embedded in the NoC at the various stages of design and fabrication. These HTs maliciously modify the type, destination, and integrity of a flit, so the proposed method is designed to detect and mitigate these HTs. These attacks will deplete bandwidth and are strongly considered in this study. The study assumes that the links of the NoC routers are to be trusted (which is guaranteed by a method presented in [38]). Because of the large area accessed by the first in first out (FIFOs) it is believed that the FIFO is more easily compromised than a submodule of the router, such as a flit processing unit. The hardware Trojan can be inserted into different parts of the network, such as the flit processing unit, the input FIFO, and FIFO. The flit processing unit is where a basic operation is performed on the flit; these operations are described below. The input FIFO is the first unit to process these flits and FIFO is used to transfer the flit through its east output port to the other routers. These units are described in Figure 5.

Figure 5 shows a general NoC on the left side of the diagram. This NoC illustrates links and routers with five ports, which are labeled west, south, east, north, and local i/o ports. These ports can accept data and send data to the NI of other routers that are attached with this router. Before the data reaches the FIFO unit, we begin by performing dynamic computation on the input side of the given circuit. This involves permuting the NoC flit bits. As a direct consequence of this, the NoC flit that is currently being held in the input of the FIFO is mixed up. Because of the dynamicity and the randomness in the permutation patterns (which are performed on the flit before entering to the input FIFO), it is difficult for a malicious attacker to exploit the content of the flit via a HT into something meaningful. We are defending our router more proactively than the router’s static protection. The underlying phenomenon of this work is to minimize the chances of the HT being inserted into the router’s FIFO, which may change the most significant bits of the NoC packet. Then, after flit permuting, we examine flit integrity, which can be destroyed via the HTs inserted at the input FIFO of the NoC router. The primary goal of this flit integrity checkup is to prevent malicious flits from entering the network, drop the flits, and then flush out the input FIFO unit if necessary. HTs may also be present at the flit processing unit as well as the FIFO’s east output port. As a result, we proposed the MAC module which presents malicious flits from entering the router. These three modules (dynamically permuting flits, flit integrity, and MAC) presented in Figure 5 provide defense against HTs entering and damaging the integrity of the flit, thereby reducing the NoC bandwidth.

**Figure 6:**
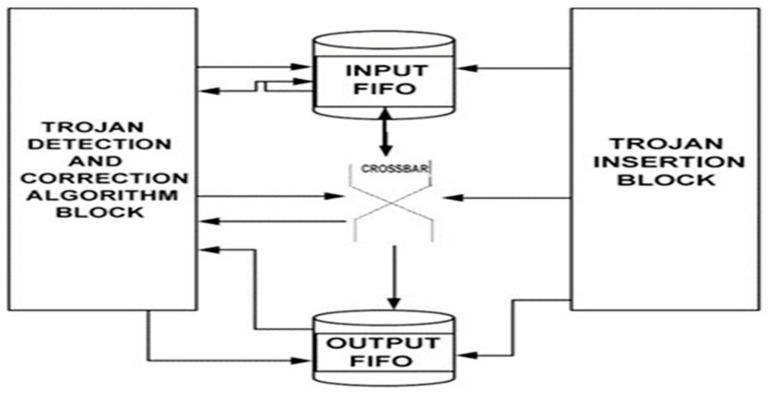
Router architecture used in this work.

Figure 6 presents a detailed explanation of the modules given in Figure 5. The critical fields, such as the destination/source address and the flits type bits, must remain unchanged to ensure that a packet reaches its intended destination. To ensure that the integrity of the flits is not changed even in the presence of HTs, we propose that the message first pass through the HMAC encoding module. Before sending the packet to the input FIFO, the next step involves applying a DFP mechanism to the buffers of the packet. This causes the bits that are regarded as being important to be reordered in a manner that is completely random. If the adversary does not know the dynamic permutation pattern, the HT that they insert will not function properly, get triggered as anticipated, or carry out the mission as it was intended. Similarly, the DFDeP operates by the side of the output port of the FIFO. Following de-permutation, the HMAC unit will regenerate another HMAC value which will be compared to the sent HMAC value to ensure the correctness of the message.

A pattern selection vector is responsible for achieving randomness in the permutation, and this randomness in the permutation is generated dynamically by a local vector generator. The random number generator, which is integrated into every router, is tasked with producing a selection vector to obtain the privateness of the flit permutation outline in favor of the dynamic flit permutation and dynamic flit de-permutation units.

The DFP routing record for dynamic flit permutation and dynamic flit de-permutation changes over time and differs from one router to another. As we know, the packet’s routing history in every router depends on the NoC. At the NoC design stage, the appliance on the operation stage and the local random generator will have a changeable output. As a result, the opponent cannot easily set any HTs that may result in successful attacks.

The permuted flits content is used in the proposed routing computation unit to determine the exact output port of the next router to which it is designed. To reduce the cost of the hardware, we use partial FDP and compare the HMAC keys to obtain exact inner and outer signals, which could be the status signals, and write requests of the FIFO buffer.

### 5.1. Proposed Technique for the Dynamic Permutation and Integrity of Flits

The proposed methodology includes an HMAC algorithm which is used to check the integrity of the data. The overall mechanism used in this paper is overviewed in Figure 6 below. In this algorithm, the critical bits, such as tail, destination address, and header field with HMAC algorithm, minimize the hardware cost and firmly control the HT inserted in the NoC. We then combine the critical bits with non-critical bits and the code word obtained from the HMAC algorithm is permuted before being stored in the FIFO buffer inputs. Then, in the routing method proposed, we partially de-permute the critical bits and the HMAC (comparing its sent key and regenerating key at this stage) to ensure that the route computation is normal. This is because biased de-permutation and HMAC key comparison can be used to obtain a valid packet output. If the integrity of the flit changes, we drop the packet and update the permutation packet.

The flit permutation technique used in this paper can prevent the flit content so that attackers are unable to quickly produce a HT. This may modify the flit content and, due to the permutation technique, the DoS attack can also be prevented. As a result, not all the critical bits can be changed by the HT and HMAC algorithms to eliminate bandwidth depletion and ensure the integrity of the flits.

**Figure 7:**
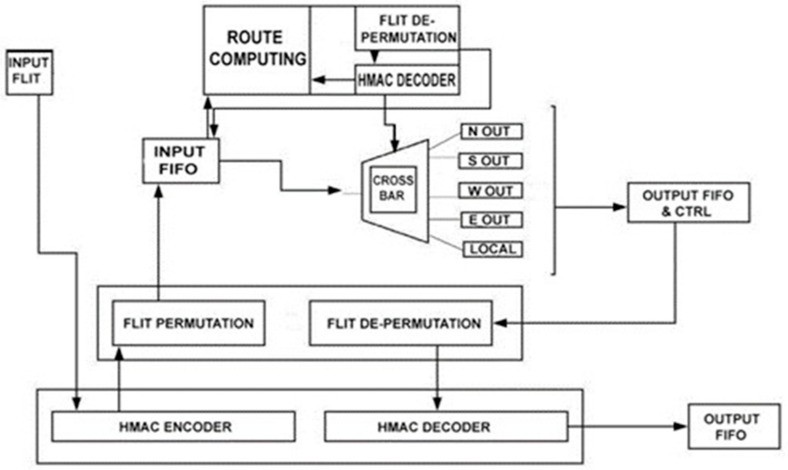
Proposed method for hardware Trojan detection and mitigation.

As shown in Figure 7, the exact process is applied to the PFDP, FDP, and FP. This is induced via the selection vector generator to further decrease transparency so that the selection vector can be applied to the five output and input ports per router. The technique presented in this study can be unmitigated, which can dynamically alter the configuration permutation if an error is detected in the field of the vital flit area. More specifically, a baseline is used to compute the route for this module, as given in Figure 7, which is presented in this work [36].

### 5.2. Dynamic PPS Update

The internal router signals and the time are relative to the PPS vector construction. When we want to make a change to the PPS vector, we will have to wait for the tail flit to arrive at its final location, which indicates that the flit has made it to its destination in its entirety. When the tail flit arrives, the input barrier checker sets the full buffer signal to high because no more buffers can enter the buffer. The signal which will become high prevents additional flits from entering the input buffers.

The network which is responsible for the generation of the PPS vector is turned on, and allows the next upcoming vector to enter the PPS vector. As the output of the buffers is emptied, evolution occurs. The evolution then directs the generation of the next clock pulse in favor of PPS vector production in order to obtain a random PPS vector by the side of the in-progress time. Then, the depressing boundary of the clock pulsate leads to alterations that twist the PPS setup and, finally, it sets the buffer signals to standard in that order.

## 6. Experimental Results

### 6.1. Experimental Setup

**Figure 8:**
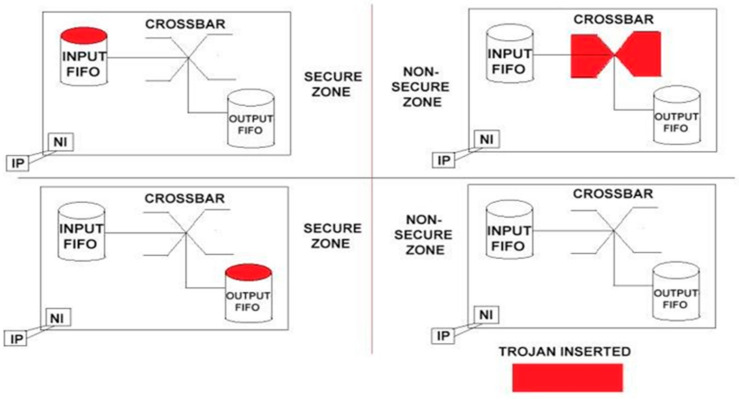
NoC with secure zone and an unsecured zone.

In the method that we have suggested, we have provided an example of a 4 by 4 mesh NoC that implements the XY routing strategy. There is use of input and output FIFOs with a depth of eight. A booksim simulator is used to perform the simulation of the system. The bookism network simulator was described by [41] as a detailed, cycle-accurate simulator for network-on-chips (NoCs) that can also be used to model interconnection networks for a variety of other systems. For the purpose of determining the directional precedence in the crossbar, a round-robin arbiter is used. In place of the random vector selection of eight distinct permutation patterns, a 3-bit PPS vector is utilized in this calculation. Figure 8 depicts the overall configuration of the NoC, which includes separate secure and unsecured zones. IP cores within a secure zone are considered trusted and authenticated, whereas those in unsecured zones may have some un-trusted modules. The packet broadcast is limited to the secure and non-secure zone under contact rules. The probability of sending and receiving packets for each protected and unprotected zone is equal. We have thoroughly calculated dissimilar packet insertion rates (λ), which vary from 0.075 en route to 0.2 packets per cycle per node.

### 6.2. Throughput

**Figure 9:**
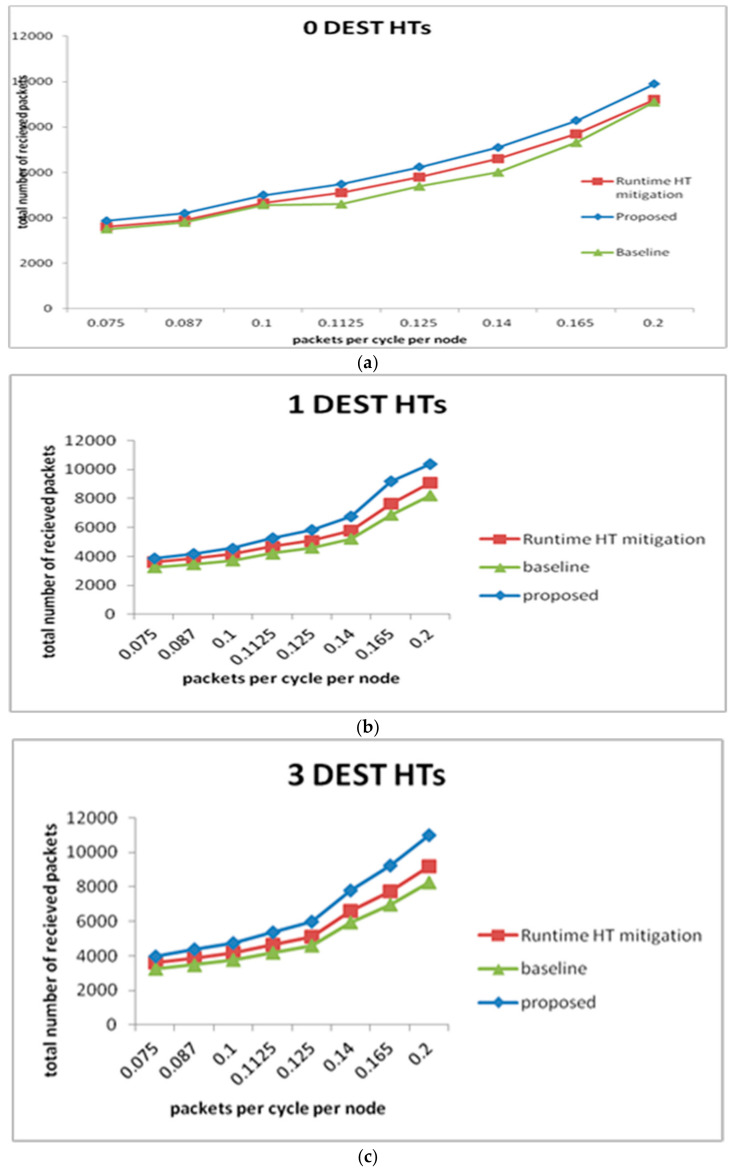
(**a**) No HT. (**b**) One dest HT. (**c**) Three dest HTs.

We compared our throughput with that of Frey et al. [21] by checking the whole quantity of received suitable packets via NoC NIs during the known simulation. The suitable packet is that which reaches its original destination somewhat quicker than that of the adapted destination address. By evaluating the performance, which is shown in Figure 9a–c, the results demonstrate that the anticipated method delivers an identical number of packets at an identical traffic vaccination rate, despite HTs being introduced. The method in [38] drops the packet as HTs are inserted. Our technique increases the number of acknowledged valid packets by up to 8% and 12% compared to the runtime hardware Trojan mitigation method and baseline, respectively, when a single HT is inserted, whereas the 3 dest HT improves some acknowledged valid packets by up to 10% compared to the runtime hardware Trojan mitigation method.

**Figure 10:**
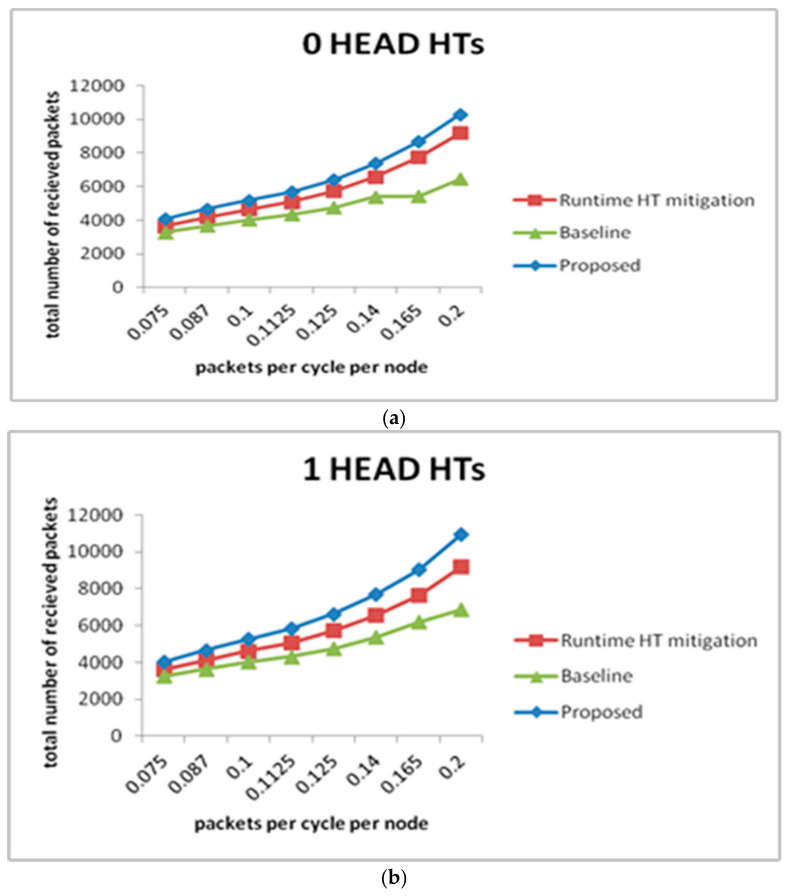

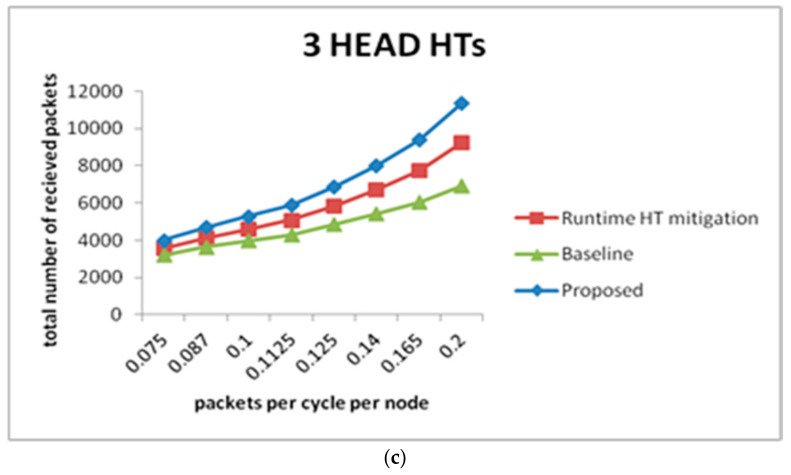
(**a**): No head HT. (**b**) One head HT. (**c**) Three head HTs.

The amount of acknowledged valid packets impacted by the HEAD HTs is shown in Figure 10. Our proposed technique also performs well in this situation because the runtime hardware Trojan mitigation method did not recover as many headers flits as our proposed method did. The number of received valid packets through our proposed method is 14.7% more than that of the runtime hardware Trojan mitigation method when 3 head HTs are inserted.

**Figure 11:**
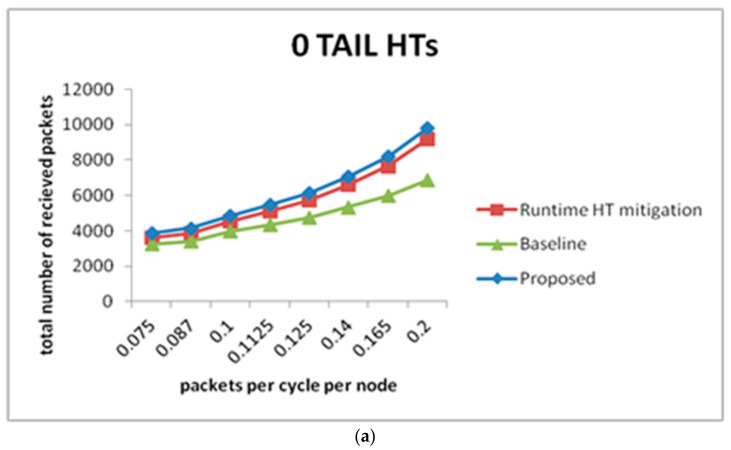

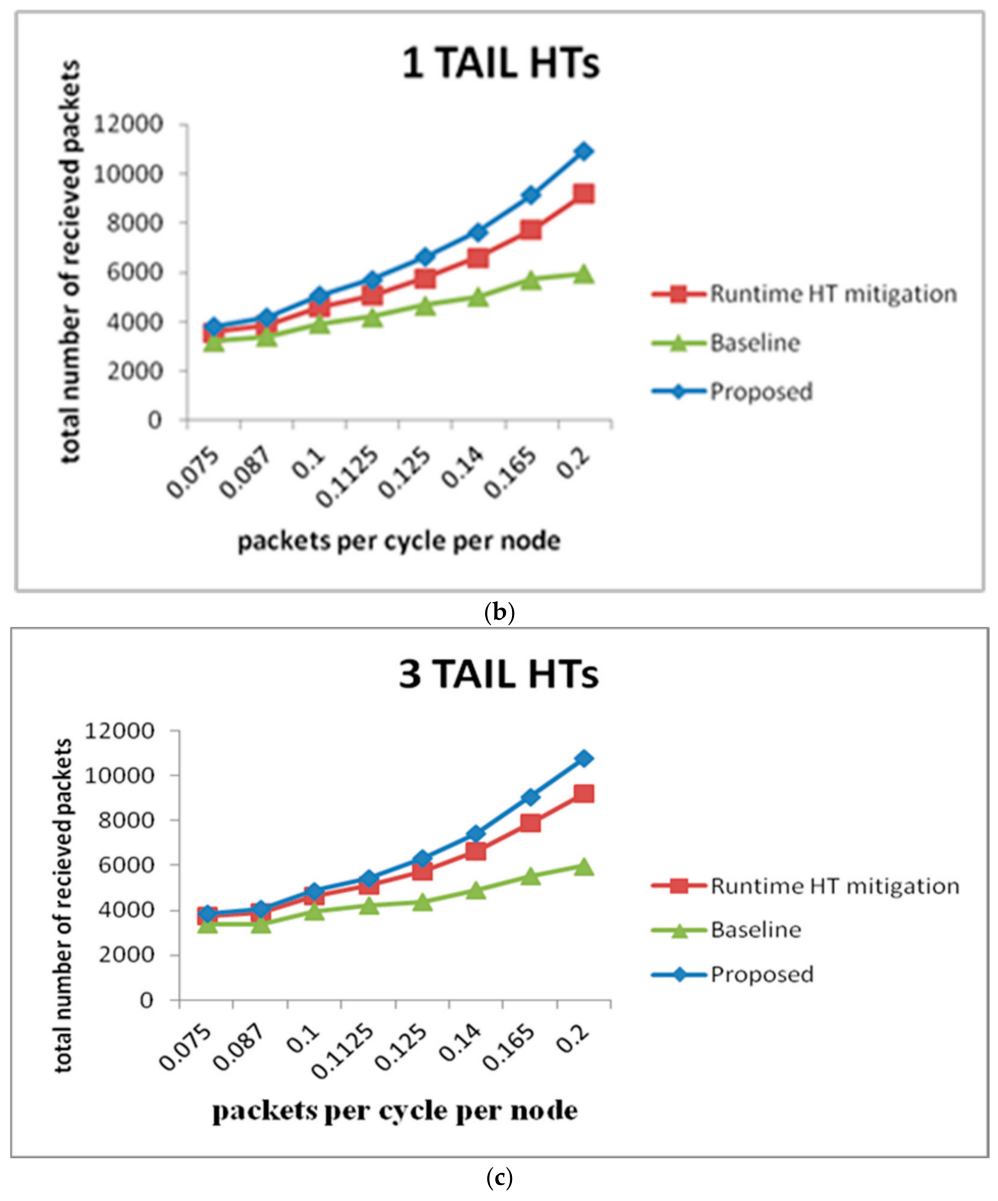
(**a**) No tail HT. (**b**) One tail HT. (**c**) Three tail HT.

The packet’s tail flit is misrouted when the TAIL HT is present. Our proposed method has a good performance compared to the runtime HT mitigation method. Our method has 3% more received valid packets than the runtime HT mitigation method, as shown in Figure 11. The performance drop due to tail flit is more than that of the HEAD HT case. This is because when the tail flit is dropped, the packets will not release the network resource, which will have more effect on bandwidth depletion.

We observe a significant change in the number of the received packets when the tail flit is lost as a result of the TAIL HT. This is in comparison to the NoC situation in which there is no HT present. As is clear from Figure 11, our method obtained exceptional results compared to the runtime hardware Trojan mitigation method, by which more valid packets were obtained when the tail HTs were inserted in the routers. However, when the number of HTs increases, there is a significant change in the throughput of our method and the runtime HT mitigation method. Figure 10b makes it abundantly clear that the decrease in performance is more detrimental due to the fact that if the tail flit does not arrive, the system will not be able to release the path through which it transfers the flits. As we increase the number of HTs being inserted, the number of routers will wait for the smash to place in the tail flits, thus preventing new packets from being inserted in the network. Therefore, it will decrease the number of valid packets obtained.

### 6.3. Migration of High-Traffic Areas and Depletion of Bandwidth

**Figure 12:**
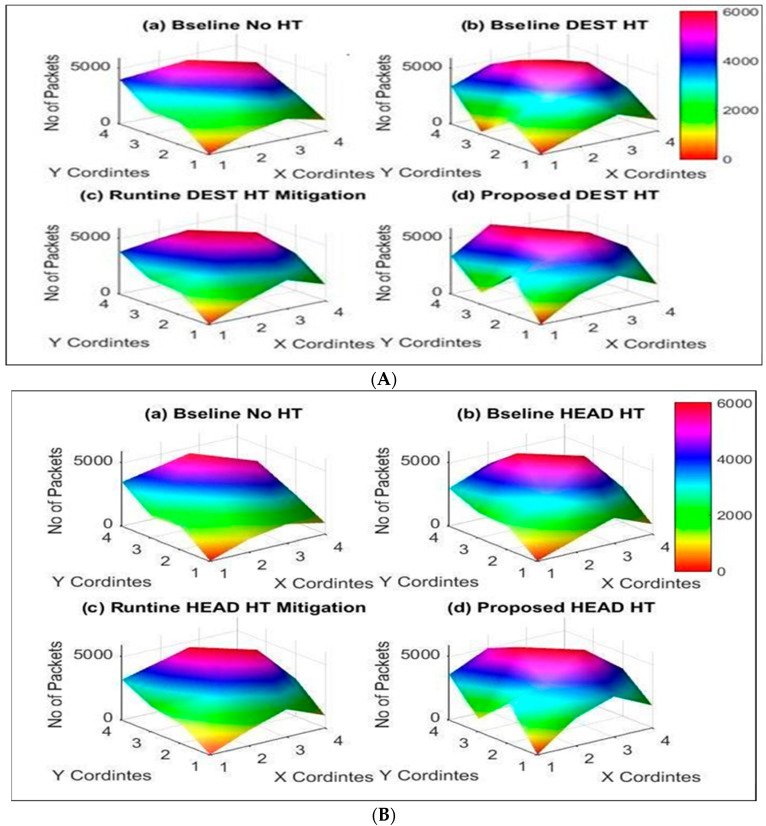
(**A**) The effect of destination HTs on traffic hotspot migration and bandwidth depletion. (**B**) The effect of destination HTs on traffic hotspot migration and bandwidth depletion.

We observed the entrance of every new flit at the output port of each router so that we could get a rough estimate of the switching task that each router was responsible for. First, the total number of flits that are sent to all of the routers is tallied, and then, using MATLAB, we use that information to create a grid with nodes, as shown in Figure 12. When a real NoC is put into operation, the switching activities of a single router are represented by a square block along with the corresponding coordinates. Throughout the entirety of the simulation process, different colors correspond to varying intensities of the number of output port switching events that take place.

Figure 12 shows switching tasks for the period of two destination HTs and two head HTs present in the NoC. We compare the result with both the baseline where no HTs are present and the runtime HTs mitigation method where two destination HTs and two head HTs are present.

Figure 12A demonstrates that the proposed method produces results that are the most comparable to the baseline, which consists of Zero HTs. Compared to the other method, the switching activity is less than that of the NI term because it transmits packets on a counter basis.

When there are two dest HTs present, the baseline NoC differs from that when there are no HTs present. This is due to the data being redirected to the incorrect local port. To illustrate this, consequently, router 1 (Y = 1 and X = 2) of the baseline NoC has a much further switching task when the two HTs are injected, compared to when no HTs are present. This is due to R1′s neighbor router, i.e., R0 (Y = 1 and X = 1), and R5 (Y = 2 and X = 2) being the vaccination points of the HTs and traffic mitigation is also observed due to the dest HTs on router R4(Y = 2 and X = 1).

In the case of head HTs, the runtime HT mitigation method has less effect on preventing the head HT, compared to our technique. As can be seen in Figure 11b, the runtime HT mitigation method receives a fewer number of packets than the baseline NoC does in situations where there is no HT.

### 6.4. Effective Average Packet Latency

**Figure 13:**
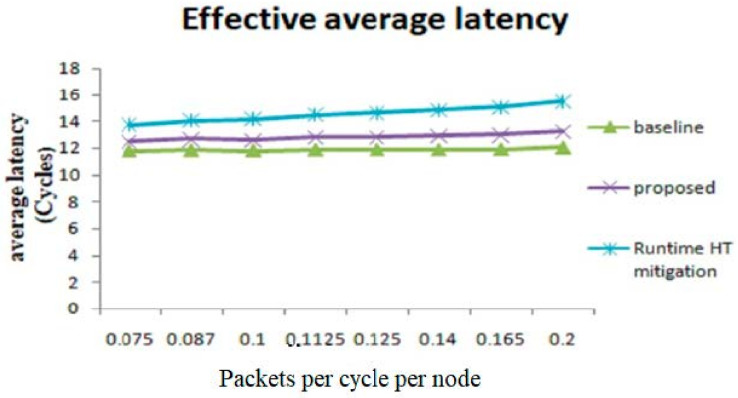
Effective average latency.

The amount of time that elapses between the point at which a packet is injected into the network and the point at which its tail arrives at the network’s destination address is referred to as latency. The average packet latency of the planned technique is shown in Figure 13, which does not change when the number of HTs increases, but varies following the increase in the injection ratio of the network.

Compared to the runtime mitigation method, our proposed method has a low *average latency*. The effective average latency is calculated by the multiplying the average latency and by the fraction of the valid packets acknowledged by the baseline when there are no HTs over valid packets unruffled by the method under test, as expressed below.

$$Effective_{\;avg\;Latency}=Avg\;\;Latency\times \frac{Valid\;Packets{\;}_{Baseline\;without\;HT}}{Valid\;Packets{\;}_{method\;under\;test}}$$

When comparing the effects of HTs on the latency, we employed the effective average latency as our metric. As can be seen in Figure 12, our proposed method reduces the effective average latency by up to 8%, 14.7%, and 3% compared to the runtime HTs mitigation method, depending on whether there are 3 dest HTs, 3 head HTs, or 3 tail HTs inserted, respectively.

## 7. Conclusions

The paper proposed a novel technique to mitigate hardware Trojan attacks in network-on-chips (NoCs) by creating secure and unsecure zones and using a PPS vector for permutation pattern selection. We evaluated the performance of our proposed technique using the booksim simulator and compared it with existing methods. Firstly, we analyzed our proposed technique’s throughput compared to the method presented in [21] and the runtime hardware Trojan mitigation method. The results showed that our technique achieved an identical number of packets at an identical traffic vaccination rate, despite the introduced hardware Trojans. Furthermore, our proposed technique increased the number of acknowledged valid packets by up to 8% and 12% over the runtime hardware Trojan mitigation method and baseline, respectively, when a single hardware Trojan was inserted. These improvements increased to 10% when three destination HTs were inserted. Secondly, we evaluated the impact of head and tail hardware Trojans on the number of acknowledged valid packets. Our proposed technique outperformed the runtime hardware Trojan mitigation method in terms of the number of received valid packets when three head HTs were inserted. However, the performance of our technique dropped by 3% when a tail HT was inserted, although it was still better than the runtime HT mitigation method. Finally, we studied the switching activity of the routers in the presence of hardware Trojans. Our proposed technique had the closest switching activity to the baseline with no hardware Trojans, while the runtime hardware Trojan mitigation method resulted in more switching activity due to its packet transmission method.

Overall, the results show that our proposed technique effectively mitigates hardware Trojan attacks in NoCs, achieving a comparable or better performance than existing methods. This study contributes to the field of hardware security and can help in the development of secure and reliable NoCs for future applications. The results indicate that the proposed hardware Trojan mitigation technique in network-on-chip (NoC) has significant potential for improving the security and reliability of NoC-based systems.

The experimental results indicate that the designed scheme can detect Trojans with a high accuracy while maintaining low overheads in terms of area, power, and performance. Therefore, this technique is applicable to the design of secure and reliable NoC systems, particularly in safety-critical applications, where the detection of hardware Trojans is crucial for system integrity.

One limitation of our method is that we consider the links between routers as trustworthy. In the future, we will assess the NoC routine by considering a method to remove this limitation.
